# Evaluation of Oxygen Radical Absorbance Capacity in Kampo Medicine

**DOI:** 10.1093/ecam/nen082

**Published:** 2011-05-26

**Authors:** Ko Nishimura, Toshihiko Osawa, Kenji Watanabe

**Affiliations:** ^1^Center for Kampo Medicine, School of Medicine Keio University, 35 Shinano-machi, Shinjuku-ku, Tokyo 160-8582, Japan; ^2^Laboratory of Food and Biodynamics, Nagoya University Graduate School of Bioagricultural Sciences, Nagoya, Japan

## Abstract

Antioxidant capacity of food has come to be shown in terms of oxygen radical absorbance capacity (ORAC) mainly on vegetables or fruit. However, the evaluation of Kampo in terms of ORAC has not yet been accomplished. It is important that such an investigation is also conducted for Kampo medicine. We measured the ORAC value of almost all the available Kampo formulas used in the Japanese National health insurance system and examined the ORAC value both for the daily prescription, and also the crude herb ingredients. The ORAC value of Kampo medicine ranged 4.65–5913 units/day. The ORAC value was high in Kampo formulas including Rhei Rhizoma, and was relatively high in Kampo formulas including anti-inflammatory herbs other than Rhei Rhizoma. The ORAC value was also high in Kampo formulas including crude herbs that have relaxation effects. The ORAC value of a crude herb would seem to not be fixed but be dependent on combination with other crude herbs from the comparison of different herbs added to the basic Kampo medicine. These results suggest variability and complexity of the antioxidant capacity of Kampo medicine within the similar range of food. On the other hand, investigation of the compound changes of various crude herbs with ORAC may lead to the elucidation of the action mechanism of Kampo medicine.

## 1. Introduction

Active oxygen is important for defending the human body against bacteria or other foreign bodies. However, when active oxygen becomes superabundant, it harms the body. Thus it is erased by antioxidants in the elimination system of the living body in order to maintain the health condition. However, in modern society, aging, carcinogenesis and arteriosclerosis are thought to be caused by production of excess active oxygen through damage by various factors such as ultraviolet rays, smoking, exhaust gas, air pollution and stress. It has become important to take food and supplements including antioxidants to prevent oxidative toxicity. Oxygen radical absorbance capacity (ORAC) is one method to measure antioxidant capacity.

In the US, 1992, ORAC was developed in National Institute on Aging by Guohua Cao [1], and it is used to analyze antioxidant capacity of various kinds of antioxidants such as catechin, flavonoid and vitamin E. This method seems to be better than others, because ORAC can measure the antioxidant capacity in both substances which are parent oily and hydrophilic, ORAC represents hydrogen atom transfer mechanism, which are most relevant to human biology, and measuring it is simple and easy [2]. On this account, ORAC has come to be used worldwide as a standard to analyze the antioxidant capacity of food and supplements scientifically. US Department of Agriculture performed the measurement of the ORAC value of plant materials on a large scale for standardization of antioxidant capacity, and showed the minute ORAC value of the various foods such as vegetables juice, sports drinks, some kinds of drink and various supplements [3].

Although it is said that Kampo medicine has antioxidant capacity examined by electron spin resonance spectrometry, several investigations have shown the capacity of only a few kinds of Kampo formulas [4, 5]. Moreover, the ORAC evaluation of Kampo medicine in itself has not been done systemically [6]. In view of the situation in which the ORAC value of food is now known, it may be important to also evaluate the antioxidant capacity of the Kampo medicine with ORAC.

## 2. Subjects and Methods

### 2.1. Subjects

Among various Kampo medicines available in Japan, 141 kinds of extract formulas, one kind of herbal powder formula and one kind of ointment formula used in the Japanese health insurance system were investigated. All medicines examined in this study were obtained from Japanese pharmaceutical companies whose manufacturing is governed by the regulations of the Pharmaceutical Affairs Law, and strictly controlled by other government regulations including Good Manufacturing Practice. As a result, products are assured of quality and safety at the highest level.

### 2.2. Measurement Method

ORAC is the direct capacity of chain-breaking antioxidant based on the hydrogen atom transfer mechanism. In this assay system, *β*-phycoerythrin (*β*-PE) was originally used as a fluorescent probe, changed to Fluorescein, 2,2′-azobis(2-amidinopropane) dihydrochloride (AAPH) as a peroxyl radical generator and 6-hydroxy-2,5,7,8-tetramethylchroman-2-carboxylic acid (Trolox, a water-soluble vitamin E analog) as a control standard. Fluorescein is added to sample or Trolox. And then, after AAPH is added, active oxygen appears and the fluorescence intensity of Fluorescein decreases. If there is any antioxidant, decrease of the fluorescence intensity is delayed. The antioxidant capacity is obtained by calculating the difference of the decrease of the fluorescence intensity between sample and Trolox. Results are expressed as ORAC units, where 1 ORAC unit equals the net protection produced by 1 *μ*M Trolox. In this study, samples were prepared in triplicate, and the results were expressed as mean.

We measured the ORAC of Kampo formulas with the method of Huang et al. [4, 5]. AAPH, Fluorescein and Trolox were completely dissolved in 75 mM disodium phosphate buffer (pH 7.4). At first, 150 *μ*L of 167 nM Fluorescein was added to each well of a 96-well polypropylene plate. After 25 *μ*L of the blank solution, 50, 25, 12.5, and 6.25 *μ*M of Trolox standard solution, and the sample solution was added to the wells, the plate was covered with a lid and incubated in the preheated (37°C) Fluorescence reader for 10 min with a 3 min shaking during this time. Then, followed by the addition of 25 *μ*L of AAPH kept in an ice bath to each well of the plate, the fluorescence was measured every 5 min for 30–45 min. As for the sample preparation, each Kampo formula (1 g) was completely dissolved in 5 mL of hot water by a supersonic wave for 2 h. Furthermore, it was mixed in vortex and centrifuged at room temperature for 3000 r.p.m., 10 min. A supernatant was collected and diluted 10 000 times in a 75 mM disodium phosphate buffer (pH 7.4) for the ORAC measurement.

### 2.3. Evaluation of ORAC Value in Kampo Medicine

ORAC is usually expressed as the capacity of 1 g in each object. However, we examined the ORAC unit for a daily dose, because doses are different for each Kampo formula.

We investigated the characteristics of Kampo formulas with high ORAC value.

Kampo formulas can be modified to form new formulas when other herbs are added to them. Comparing the ORAC values between the basic and the modified formulas, we can examine the characteristics of the additional herbs.

## 3. Results

### 3.1. ORAC Value of Kampo Formula Per Daily Dose

When we calculated the ORAC value of Kampo formula per daily dose, it ranged from 4.65 to 5913.

### 3.2. Characteristics of Kampo Formulas in that ORAC Value Was High

The ORAC value of Kampo formulas containing Rhei Rhizoma (junchoto, tokakujokito, bofutsushosan, choijokito, daiokanzoto, jizusoippo, tsudosan, san'oshashinto, mashiningan, daijokito, keishikashakuyakudaioto, inchinkoto) was high (Table 1).

The ORAC value of Kampo formulas containing anti-inflammatory herbs other than Rhei Rhizoma (saikokeishikankyoto, keigairengyoto, seijobofuto, jinsoin,nyoshinsan, kososan, saikanto, saikoseikanto, chikujountan-to, saibokuto, san'oshashinto, senkyuchachosan, inchinkoto) was also high (Table 1).

The ORAC value of Kampo formulas that relax the muscles (kakkonto, kakkontokasenkyushin'i, jinsoin, saibokuto, shomakakkonto, tsudosan, daijokito, mashiningan) was relatively high (Table 1).

The ORAC value was extremely high in tsudosan, mashiningan, daijokito. For these Kampo medicines, Magnoliae Cortex for relaxing muscle tonus is included rather than Rhei Rhizoma. In other words, the more a Kampo medicine contains herbs of a high ORAC value, the higher the overall ORAC value (Table 1).

### 3.3. Difference of the ORAC Value of Kampo Formulas According to the Additional Herbs Added to the Basic Kampo Formula

**Figure 1:**
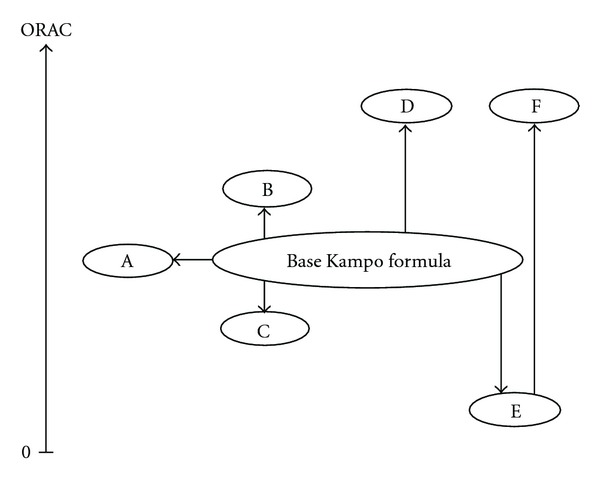
Difference of the ORAC value of Kampo formulas according to the additional herbs added to the basic Kampo formula. Major change patterns were classified into six groups in this study. Group A, which had little change of ORAC value, consisted of shokenchuto and tokikenchuto shown in Table 2, and keishikajutsubuto and keishikaryojutsubuto shown in Table 4, respectively. Group B, which had mild increase of ORAC value compared to base Kampo formula, consisted of ogikenchuto shown in Table 2, and tokishigyakukagoshuyushokyoto shown in Table 4, respectively. Group C, which had mild decrease of ORAC value compared to base Kampo formula, consisted of hachimijiogan and goshajinkigan shown in Table 3, and keishikaryukotsuboreito and keishikakobokukyoninto shown in Table 4, respectively. Group D, which had high increase of ORAC value compared to base Kampo formula, consisted of keishikashakuyakudaioto shown in Table 2. Group E, which had high decrease of ORAC value compared to base Kampo formula, consisted of keishikaogito and keishikakakkonto shown in Table 4. Group F, which had much high increase of ORAC value compared to Group E, consisted of kakkonto and kakkontokasenkyushin'i; shown in Table 4.

In the Kampo formula series based on keishikashakuyakuto, it was recognized that Rhei Rhizoma has an influence on high antioxidant capacity, which Paeoniae Radix, Astragali Radix, Angelicae Radix, Saccharum Granorum did not have (Table 2 and Figure 1).

In the Kampo formula series based on rokumigan, it was found that Cinnamomi Cortex, Aconiti Tuber, Achyranthis Radix, Plantaginis Semen did not have an influence on antioxidant capacity (Table 3 and Figure 1).

In the Kampo formula series based on keishito, strong antioxidant capacity was not recognized by the addition of Fossilia Ossis Mastodi, Ostreae Testa, Atractylodis Lanceae Rhizoma, Aconiti Tuber, Astragali Radix. Additional Magnoliae Cortex or Puerariae Radix deteriorated the antioxidant capacity. However, strong antioxidant capacity was identified by addition of the combination of Ephedrae Herba and Puerariae Radix. The addition of Evodiae Fructus, Angelicae Radix, Akebiae Caulis and Asiasari Radix showed little antioxidant capacity (Table 4 and Figure 1).

## 4. Discussion

The present study showed following results. The ORAC value in a daily dose of Kampo formula in Japan was 4.65–5913. The ORAC value in the dose was high in Kampo formulas including Rhei Rhizoma, and was relatively high in Kampo formula including anti-inflammatory herbs other than Rhei Rhizoma. The ORAC value was also high in Kampo formula including crude herbs that have relaxation effects. The change in antioxidant capacity from the addition of a crude herb varied with different base formulas.

Although it is said that Kampo medicine has high antioxidant capacity, the ORAC value taken in on one day by Kampo medicine did not seem to be high compared to a meal judging from this ORAC measurement results. Actually, the quantity of ORAC which we can take in with food (vegetables, fruit) on one day is 3264 ORAC units in total. This referred to the National Health and Nutrition Survey in Japan [7] and calculated by the ORAC measurements data of each food [3]. When we convert an intake of each food into ORAC value, that of fruit was 1182 ORAC units, that of vegetables was 2082 ORAC units. In terms of ORAC value, the similarity between Kampo medicine and food may partially be explained by the fact that Kampo medicine consists of herbs. However, antioxidant effects of Kampo medicine should be evaluated *in vivo*. Moreover, the ORAC method has a weakness that it cannot measure antioxidant capacity of carotenoid because the methodological principle is based on the mechanism of hydrogen migration. It is necessary to examine consistency with the antioxidant capacity measured by other *in vitro* methods. DPPH (1,1-diphenyl-2-picrylhydrazyl) method, FRAP (Ferric Reducing Ability of Plasma) method and TRAP (Total Radical-trapping Antioxidant Parameter) method are well known [2], and they should be compared to ORAC value. Moreover, we should discuss the absorptive efficiency of Kampo medicine. However, it is not well known, although pharmacokinetics of some of compounds was investigated in clinical and basic researches [8, 9].

Because Rhei Rhizoma, a component herb in many Kampo formulas, has anti-inflammatory action, and anti-tumor effect, it may be possible that the ORAC value of Kampo formulas including Rhei Rhizoma is high. For example, in examination using KHC rabbit, which is a model of the familial hypercholesterolemia, the development of arteriosclerosis was inhibited by the dosage of choijokito of 1 g kg^−1^ of 24 weeks [10]. Although daisaikoto did not improve hypercholesterolemia in KHC rabbits, it restrained the development of arteriosclerosis in the aorta and showed an antioxidant capacity for the LDL [11]. In addition, in the study using the same model, san'oshashinto made the use rate of *α*-tocopherol high, and had antioxidant capacity for the LDL, although it did not give a change to serum lipid [12]. In addition, Rhyu et al. [13] reported that Rhei Rhizoma had the highest antioxidant capacity among constitution crude herbs in Wen-Pi-tang. These findings suggest the *in vitro* antioxidant capacity of Kampo formulas including Rhei Rhizoma by ORAC measurement was supported even *in vivo*.

Kampo formulas which contain herbs that have anti-inflammation action except Rhei Rhizoma had high ORAC value, which were not as high as that of Rhei Rhizoma. Judging from the crude herb constitution of a high ORAC value Kampo formula, Coptidis Rhizoma, Scutellariae Radix, Bupleuri Radix, Gardeniae Fructus, Phellodendri Cortex, Forsythiae Fructus, Arctii Fructus, and Menthae Herba are thought to be candidates as anti-inflammatory herb. Kampo formulas that have anti-inflammatory effect are generally classified to two groups, one is the group that contains Bupleuri Radix and Scutellariae Radix, and the other is the group that contains Coptidis Rhizoma, Scutellariae Radix, Phellodendri Cortex and Asiasari Radix, in addition to Kampo formulas containing Rhei Rhizoma. And the investigations about the antioxidant capacity have been accomplished according to such a classification. For example, regarding the former group, Sakaguchi et al. [14] reported the potential for protection of hepatic cells from free radicals in endotoxemia. Sakaguchi et al. [15] reported that shosaikoto controlled nitric oxide in the macrophage induced by endotoxin. Inoue et al. [16] showed shosaikoto inhibited arteriosclerosis by improving a decrease of nitric oxide of a macrophage caused by high lipid food intake. Egashira et al. [17] reported that shosaikoto did scavange superoxide anion radicals, hydroxyl radicals, 1,1-diphenyl-2-picrylhydrazyl radicals on dose-dependence of the formula. In the view that saireito prohibited an increase of mesangial cells in mesangioproliferative glomerulonephritis, Liu et al. [18] considered that this was based on antioxidant effect. Ohta et al. [19] reported that shigyakusan inhibited the acute gastric mucosal lesion caused by compound 48/80, which was based on its restoration action to increased infiltration of neutrophile, sthenia of lipid peroxidation and failure of defensive function of gastric mucosa. On the other hand, regarding the latter group, Sekiya et al. [20] reported that orengedokuto restrained the arteriosclerosis of the rabbit with hypercholesterolemia. Sakuma et al. [21] reported that shichimotsukokato seemed to inhibit hypertension and arteriosclerosis by raising the serum level of nitrogen oxide at the level that does not cause harmful side effect. Akamatsu et al. [22] reported that the efficacy of keigairengyoto for Verruca vulgaris was based on the antioxidant effect on neutrophiles infiltrating Verruca vulgaris. Stefek and Benes [23] reported that orengedokuto decreased free radical 1,1-diphenyl-2-picrylhydrazyl. In addition, Nakajima et al. [24] and Hamada et al. [25] reported that baicalein which is constitution ingredient of Scutellariae Radix suppressed the increase in the thiobarbituric acid-reactive substances in the brain of rats with FeCl_3_-induced epilepsy, and also inhibited hippocampal neuronal death in gerbils with transient ischemia.

In Kampo formulas including crude herbs such as Magnoliae Cortex, Puerariae Radix, which have muscle relaxation effects, ORAC measurement showed a high value in this investigation. And there are some reports to suggest antioxidant effect of these medicines. About Magnoliae Cortex, Son et al. [26] reported that magnolol and honokiol inhibited production of iNOS and THF-*α* in the experiment system using RAW264.7 cell. Chiu et al. [27], Haraguchi et al. [28] and Taira et al. [29] reported that magnolol and honokiol eliminates hydroxyl radical, and inhibited lipid hyperoxidation, resulting in prevention of liver damage by the peroxide in rats. Because a fall of cytochrome P450 (CYP) content and CYP2E1-dependent *p*-nitrophenol hydroxylase activity were found in liver microsome of Wister rat treated with Puerariae Radix, Speroni et al. [30] pointed out the antioxidant capacity of Puerariae Radix. In addition, Kang et al. [31] reported that Puerariae Radix inhibited oxidative stress induced by hydrogen peroxide or streptozotocin. However, having found poor ORAC values in Kampo formulas without Rhei Rhizoma, we cannot expect high antioxidant capacity in Kampo formulas with only herbs that have muscle tonus relaxation effect.

Our data suggest that the effects of Kampo medicine come from the composition and interaction of the ingredients during the boiling process, and not simply the aggregate effects of each individual component. In other words, the effect of the added crude herb is not a simple extension of the base formula characteristic; rather the addition of a crude herb creates a new Kampo formula with new characteristics. When we compared rokumigan as hachimijiogan and goshajinkigan, which contain additional Cinnamomi Cortex and Aconiti Tuber to rokumigan, the ORAC value of hachimijiogan and goshajinkigan, the new formulas were lower than that of rokumigan. On the other hand, when we compared keishito as keishikajutsubuto, the formula to which Atractylodis Lanceae Rhizoma and Aconiti Tuber are added, the ORAC value of keishito was the same as that of keishikajutsubuto. These findings may show discrepancy in the function of Aconiti Tuber. The ORAC value of keishikakakkonto, which contains additional Puerariae Radix to keisihito, was lower than that of keisihito. The ORAC value of kakkonto, which contains additional Puerariae Radix and Ephedrae Herba to keisihito, was higher than that of keisihito. These finding may suggest that Kampo medicine has complicated characteristics that cannot be assumed from each simple herb. And it is expected that the functions of herbs might be clarified by investigating changes of complicated herbal formulas with the ORAC method.

In this discussion, the antioxidant capacity of Kampo formulas was confirmed both *in vitro* and *in vivo* in the previous reports. However, sometimes the discrepancy exists between *in vivo* and *in vitro* results. For example, although ORAC unit of keishibukuryogan was not so high, the antioxidant capacity was observed *in vivo* [32]. Moreover, there are several problems in the investigations of the antioxidant capacity of Kampo medicine. There was no significant correlation between ORAC values and nature or functions of some herbs [33]. Some Kampo formula and its individual herb components varied in their ORAC values [6]. The ORAC evaluation of Kampo herbs has not been done systemically [34]. It seems to be necessary to investigate the relationship of ORAC values between Kampo formula and the crude herbs. Combination of the integrated ORAC data *in vitro* and the results obtained from investigations *in vivo* by reliable methods will lead the accurate antioxidant capacity of Kampo medicine.

## Funding

Special Co-ordination Fund for Promoting Science and Technology.

## Figures and Tables

**Table 1:** The 50 highest ORAC value Kampo formulas.

Kampo formula	ORAC		
	Unit (extact)	Unit (formula)	Unit (formula/ day)
tsudosan	1314.06	788.44	5913.27
daijokito	1825.25	730.10	5475.76
mashiningan	2387.72	716.32	5372.37
daiokanzoto	2919.12	583.82	4378.69
tokiinshi	867.44	578.29	4337.19
daisaikotokyodaio	759.70	481.14	4330.30
junchoto	855.02	570.01	4275.11
keigairengyoto	930.50	558.30	4187.25
saikoseikanto	879.31	556.90	4176.74
kakkonkajutsubuto	802.34	513.50	3851.23
chikujountanto	672.94	493.49	3701.18
kakkontokasenkyushin'i	920.78	491.08	3683.11
san'oshashinto	2100.18	490.04	3675.31
bofutsushosan	798.67	479.20	3594.02
seijobofuto	753.58	477.27	3579.52
tokakujokito	1179.57	471.83	3538.71
choijokito	2830.01	471.67	3537.52
kakkonto	925.17	462.58	3469.38
keishikashakuyakudaioto	856.27	456.68	3425.09
jidabokuippo	1512.87	453.86	3403.95
daisaikoto	755.43	453.26	3399.43
kososan	1695.17	452.04	3390.33
shomakakkonto	1505.41	451.62	3387.18
saikanto	676.92	451.28	3384.61
nyoshinsan	749.25	449.55	3371.63
senkyuchachosan	1024.73	444.05	3330.37
inchinkoto	2218.58	443.72	3327.86
saibokuto	653.80	435.87	3269.02
yokukansankachimpihange	700.31	420.19	3151.40
saikokeishikankyoto	868.91	405.49	3041.18
jinsoin	758.19	404.37	3032.76
shin'iseihaito	666.29	399.77	2998.28
shinpito	1089.68	399.55	2996.61
boiogito	788.37	394.19	2956.40
nijutsuto	582.32	388.22	2911.62
seishinrenshiin	579.39	386.26	2896.93
otsujito	723.38	385.81	2893.54
hangeshashinto	642.44	385.46	2890.96
ogonto	715.29	381.49	2861.18
heiisan	863.41	374.15	2806.09
orengedokuto	1847.38	369.48	2771.07
tokishigyakukagoshuyushokyoto	679.20	362.24	2716.78
gorinsan	542.33	361.55	2711.66
kyukikyogaito	440.86	293.91	2645.16
jizusoippo	866.79	346.72	2600.38
daiobotanpito	742.25	346.38	2597.87
saikokaryukotsuboreito	567.35	340.41	2553.07
shofusan	634.90	338.62	2539.62
goshakusan	631.23	336.66	2524.91
bukuryoingohangekobokuto	559.71	335.83	2518.72

**Table 2:** The ORAC value of Kampo formulas according to the additional herbs added to keishikashakuyakuto (base formula).

Kampo formula	Additional herbs added to base formula	ORAC value of Kampo formula per daily dose (*μ*mol TE)
keishikashakuyakuto		1796.7
keishikashakuyakutd aioto	Rhei Rhizoma	3425.1
shokenchuto	Saccharum Granorum	1840.2
ogikenchuto	Saccharum Granorum + Astragali Radix	2174.04
tokikenchuto	Angelicae Radix	1923

**Table 3:** The ORAC value of Kampo formulas according to the additional herbs added to rokumigan (base formula).

Kampo formula	Additional herbs added to base formula	ORAC value of Kampo formula per daily dose (*μ*mol TE)
rokumigan		1842.98
hachimijiogan	Cinnamomi Cortex+ Aconiti Tuber	1050
goshajinkigan	Cinnamomi Cortex + Aconiti Tuber+ Achyranthis Radix + Plantaginis Semen	1097.03

**Table 4:** The ORAC value of Kampo formulas according to the additional herbs added to keishito (base formula).

Kampo formula	Additional herbs added to base formula	ORAC value of Kampo formula per daily dose (*μ*mol TE)
keishito		1804.28
keishikaryukotsuboreito	Fossilia Ossis Mastodi+Ostreae Testa	1246.65
keishikajutsubuto	Atractylodis Lanceae Rhizoma + Aconiti Tuber	1751.1
keishikaryojutsubuto	Atractylodis Lanceae Rhizoma + Aconiti Tuber + Poria	1710.15
keishikaogito	Astragali Radix	538.2
keishikakakkonto	Puerariae Radix	609.9
keishikakobokukyoninto	Magnoliae Cortex + Armeniacae Semen	1374.45
kakkonto	Puerariae Radix + Ephedrae Herba	3469.35
kakkontokasenkyushin'i	Puerariae Radix + Ephedrae Herba + Cnidii Rhizoma + Magnoliae Flos	3683.1
tokishigyakukagosh uyushokyoto	Angelicae Radix + Evodiae Fructus + Akebiae Caulis + Asiasari Radix	2716.8
keishikashakuyakuto	Paeoniae Radix^a^	1796.7

^a^Keishikashakuyakuto contains more Paeoniae Radix than keishito.
